# Supplementary material for: Micronutrient Analysis of Gluten-Free Products: Their Low Content Is Not Involved in Gluten-Free Diet Imbalance in a Cohort of Celiac Children and Adolescent
Source: Foods. 2019 Aug 7;8(8):321. doi: 10.3390/foods8080321 (PMC6723272; doi:10.3390/foods8080321)
Supplement: Supplementary file 1 [file foods-08-00321-s001.pdf]

Supplementary Materials

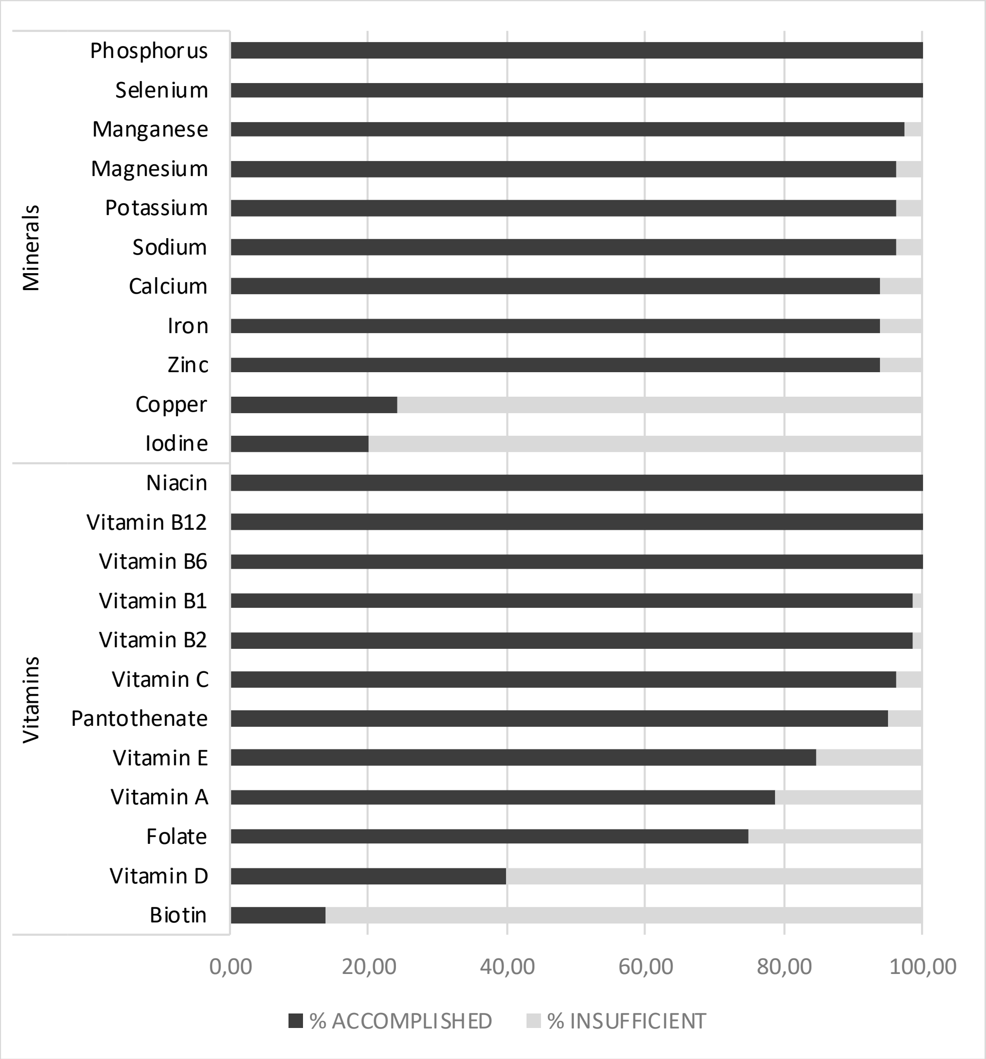

Figure S1. Percentage of celiac children and adolescents who accomplished or did not achieve 2/3 of the dietary reference intake of the vitamins and minerals (proposed by the Federation of Spanish Societies of Nutrition and Dietetics, FESNAD).

Table S1. Analyzed gluten free products and ingredients declared on the package label.

Code	Product	Ingredients
Cereal Flakes 1	Choco balls	Corn semolina 61%, whole cane sugar, bitter cocoa 8% (cocoa dough, whole cane sugar, cocoa butter), cocoa powder 5%, rice flour 4%, quinua flour 4%, sea salt
Cereal Flakes 2	Granola choccolate and almonds	Buckwheat and millet flakes 30%, sugar, rice flour, vegetable oil, corn flakes (corn flour, salt), chocolate 5% (sugar, cocoa paste, cocoa butter, emulsifier: soy lecithin, flavor), raisins (raisin, vegetable oil), puffed rice, coconut 3.5%, potato starch, almonds 2%, cocoa, antioxidant: ascorbic palmitate, tocopherols (natural), soy lecithin
Cereal Flakes 3	Milly Magic	Corn semolina, sugar, cocoa powder 8%, iodized salt
Cereal Flakes 4	Choco bites	Cereals (54.6%) (whole rice flour, corn flour), sugar, skimmed cocoa powder (6.3%), rapeseed refined oil, hazelnuts (2.1%), lactose, palm oil, whey powder, sunflower refined oil, skimmed milk powder, emulsifier (sunflower lecithin), flavor
Cereal Flakes 5	Corn Flakes	Corn, sugar, salt, emulsifier: sunflower lecithin, vitamins: niacin, pantothenic acid, B6, riboflavin, thiamine, folic acid, biotin, B12
Cereal Flakes 6	Corn Flakes	Corn, sugar, salt and corn glucose
Cereal Flakes 7	Corn Flakes	Corn (98.6%), sugar, salt, dextrose, partially inverted whole sugar syrup, acidity regulator
Cereal Flakes 8	Corn Flakes	Corn, sugar and salt.
Cereal Flakes 9	Corn Flakes	Corn 90%, whole cane sugar, rice syrup, sea salt
Cereal Flakes 10	Cereals, cocoa and honey	Corn semolina (72%), sugar, honey (2.8%), cocoa (powder), dextrin, salt, vanilla
Cereal Flakes 11	Corn Flakes	Corn 93.4%, sugar, salt, emulsifier: sunflower lecithin, vitamins: niacin, pantothenic acid, B6, B2, B1, folic acid, biotin and B12
Cereal Flakes 12	Choco bubbles	Corn flour 63%, sugar, cocoa, vegetable oil, emulsifier: soy lecithin, and flavors
Cereal Flakes 13	Honey Balls	Corn flour, sugar, honey 3%, sunflower oil, salt
Bread 1	Rustic bread	Corn starch, water, rice flour, sugar, sunflower oil, stabilizers, corn fiber, yeast, salt, raising agent (sodium bicarbonate), emulsifiers, preservative, flavors, antioxidant
Bread 2	Breadmaking preparation	Corn starch, sugar, stabilizer (guar gum and hydroxypropyl methyl cellulose), salt and raising agent (sodium bicarbonate)
Bread 3	Breadmaking Mix	Corn starch, flax seed flour 12%, buckwheat flour 8%, pea bran, rice bran, apple fiber, sugar, thickener: guar gum, salt
Bread 4	Vienés baguette	Water, corn starch, margarine, water, emulsifier, acidifier, preservative, flavors, food colour, sugar, tapioca starch, rice flour, thickener, yeast, emulsifier, rice fiber, salt, preservative, raising agents, antioxidant, flavors
Bread 5	Sliced bread	Corn starch, dextrose, thickener, emulsifier, salt, millet flour, raising agent, preservative, antioxidant, bamboo fiber, yeast, sunflower oil, moisturizing and water
Bread 6	Fresh bread	Water, corn starch, sourdough, rice starch, cereals, apple fiber, beet syrup, sunflower oil, rice syrup, soy flakes 2.1%, sunflower seeds 2,1%, soy bran 1.9%, flax seeds 1.9%, thickeners, millet flakes 1.4%, soy protein, yeast, sea salt, honey
Bread 7	Precooked baguettes	Corn starch 66%, water, sugar, thickener (xanthan gum), emulsifier (monoacetyl and diacetyl tartaric esters of monoglycerides and diglycerides of fatty acids), yeast, salt, preservative (calcium propionate), antioxidant (ascorbic acid), raising agents (disodium diphosphate , sodium bicarbonate)
Bread 8	Gluten free breadcrumbs	Corn starch 49%, water, pasteurized liquid egg, sugar, emulsifiers (monoglycerides and diglycerides of fatty acids, monoacetyl and diacetyl tartaric esters of monoglycerides and diglycerides of fatty acids), salt,

		thickener (xanthan gum), raising agents (disodium diphosphate, sodium bicarbonate)
Bread 9	Baguette	Corn starch, sourdough 28% (rice flour, water), water, rice syrup, apple fiber, rice starch, sunflower oil, soy protein, sugar, thickener: hydroxypropyl methyl cellulose, yeast, salt
Bread 10	Toasts	Corn starch, dextrose, thickener (E-412; E-415; E-466), emulsifier (E-471; E-472e), salt, millet flour, raising agent (E-500ii; E-E-541i; E-341), preservative (E-200), antioxidant (E-300), bamboo fiber, yeast, sunflower oil, moisturizing (glicerol) and water
Bread 11	Bread roll	Corn starch, vegetable margarine, sugar, salt, water, thickener, emulsifier, raising agent, antioxidant, preservative and yeast.
Bread 12	Crispy toasts	Corn starch, water, sugar, pasteurized liquid egg, margarine, sunflower oil, water, emulsifier, acidity regulator, preservative, flavors, yeast, thickener, emulsifier, salt, preservative, antioxidant, raising agents
Pasta 1	Pasta	Corn flour and water
Pasta 2	Rice and corn Penne Pasta	Rice flour 87.7%, corn flour 12.3%
Pasta 3	Corn macaroni propellers with vegetables	Corn flour (55%), rice flour (41.5%), quinua flour (3%), emulsifier E-471 (mono and diglycerides of fatty acids). Pasta with tomato: corn flour (53.5%), rice flour (39.5%), tomato concentrate (5%), quinua flour (2%), emulsifier E-471 (mono and diglycerides of fatty acids). Pasta with spinach: corn flour (54.5%), rice flour (41%), quinua flour (2.5%), dehydrated spinach (2%), emulsifier E-471 (mono and diglycerides of fatty acids)
Pasta 4	Pasta for Kids	Corn flour and water
Pasta 5	Penne	Corn flour, millet flour, rice flour, sugar cane syrup
Pasta 6	Rice noodles	Rice flour and corn starch
Pasta 7	Corn and Rice Pasta shapes	Corn flour 79.2%, rice flour 19.8%, soy lecithin
Pasta 8	Lasagne mini sheets	Rice flour 87.3%, corn flour 12.7%
Pasta 9	Pasta all uovo	Corn starch, eggs 23%, rice flour, corn flour, emulsifier (E471), thickener (guar gum)
Pasta 10	Whole rice macaroni	Whole rice flour* (98%), thickener: xanthan gum. *From organic farming
Pasta 11	Rigatoni	Corn starch, pre-treated corn flour, rice flour, rice starch, pea proteins (5%), lupine flour (2%), emulsifier: mono and diglycerides of fatty acids
Pasta 12	Meat cannelloni	Dough 55%, corn starch, egg powder, glucose syrup, sugar, stabilizers, salt and water + meat filling
